# Eccentric Hamstring Muscle Strength during Home Confinement Due to the COVID-19 Pandemic, and Football Competition Resumption in Professional Football Referees: A Prospective Observational Study

**DOI:** 10.3390/ijerph18189737

**Published:** 2021-09-15

**Authors:** Víctor Moreno-Pérez, Marc Madruga-Parera, Daniel Romero-Rodríguez, Javier Sanchéz-Sanchéz, José Luis Felipe, Lluis Marcè-Hernández, Eudald Recasens-Sarrà, Juan Del Coso

**Affiliations:** 1 Sports Research Center, Miguel Hernandez University of Elche, 03202 Alicante, Spain; vmoreno@umh.es; 2 Center for Translational Research in Physiotherapy, Department of Pathology and Surgery, Miguel Hernandez University of Elche, 03202 Elche, Spain; 3 Physical Therapy Department, Universitat Internacional de Catalunya, 08017 Barcelona, Spain; marcmparera@gmail.com (M.M.-P.); danirrphysco@gmail.com (D.R.-R.); 4 FC Barcelona Second Team, Sport Performance Area, 08028 Barcelona, Spain; 5 reQ, Return to Play and Sports Training Center, 08028 Barcelona, Spain; lluispfm@gmail.com (L.M.-H.); eudaldrecasens.cvb@gmail.com (E.R.-S.); 6 FC Barcelona First Team, Sport Performance Area, 08028 Barcelona, Spain; 7 Faculty of Sport Sciences, Universidad Europea de Madrid, 28670 Madrid, Spain; javier.sanchez2@universidadeuropea.es (J.S.-S.); joseluis.felipe@universidadeuropea.es (J.L.F.); 8 Comité Técnico de Árbitros (CTA), Real Federación Española de Fútbol, 28230 Las Rozas, Spain; 9 Centre for Sport Studies, Rey Juan Carlos University, 28943 Fuenlabrada, Spain

**Keywords:** strength, injury risk, muscle performance, exercise performance, muscle weakness

## Abstract

The COVID-19 pandemic has produced a major disruption for professional football leagues that has affected the physical preparation of both football players and referees. In Spain, health authorities decreed home confinement for eight weeks, supressing the normal training routines of professional referees. After home confinement, referees had four weeks to retrain as the national football league was set to resume matches to complete the 11 games remaining. The aim of the present investigation was to assess changes in eccentric hamstring muscle strength during football competition suspension/resumption due to the COVID-19 pandemic in 21 professional football referees (mean ± SD, age: 33.4 ± 5.1 years; height: 182.4 ± 5.0 cm; body mass: 75.1 ± 4.4 kg). Eccentric hamstring muscle strength was measured with the Nordic hamstring exercise at four time points. During home confinement, referees presented the lowest value of bilateral eccentric muscle strength (300 ± 14 N). Eccentric muscle strength increased by 13.2 ± 3.7% one week after the end of home confinement (339 ± 16 N; *p* = 0.001, effect size (ES) = 2.8) and remained stable before the first match (343 ± 17 N; *p* = 0.001, ES = 3.1) and after the end of the national league (328 ± 13 N; *p* = 0.001, ES = 2.0). In summary, home confinement produced detraining effects in professional football referees associated with hamstring muscle weakness. In this regard, strength-based activities with body loads may be insufficient to avoid muscle weakness and other means (e.g., weights) may be necessary to maintain muscle strength. However, the 4-weeks retraining period was sufficient to resolve hamstring muscle weakness induced by the restrictions of home confinement. This information may be helpful in the case of future sport competition suspension or home quarantine due to new waves of COVID-19 pandemic.

## 1. Introduction

Several investigations have described the external physical demands of professional football (soccer) refereeing during an official match [1,2,3,4]. Referees cover between 10 and 12 km during matches and approximately 18.6% of this distance is performed at high-intensity (running speeds from 15 to 25 km/h) [1], while these physical demands can be higher in matches including top level teams [5]. This running distance can only be obtained with maximal oxygen uptake above 50 mL/kg/min and a running speed at anaerobic threshold of ~14 km/h [6]. In addition, professional football referees perform almost 40 sprints per match at running speeds above 18 km/h [2]. To this, must be added the cognitive load of refereeing, with between 104 and 162 observable decisions made in each match [7]. Previous reports have shown an incidence of between 2.16 and 20.8 injuries per 1000 h [8,9,10] due to the high physical demands of professional refereeing in football, and the injury risk is higher for match officiating compared to training [11,12]. Specifically, strains are the most common type of injury and the hamstrings are one of the commonest body locations for the muscle strains suffered in professional referees [9,10,12]. Referees, and their two assistant referees, play a key role in the game of football, obliging them to follow the match closely to detect infractions. The importance of the physical readiness of top-class match officials and the limited number of referees in most national leagues suggests the importance of establishing injury prevention programmes for professional football referees, as has been the case for professional football players [13]. In this regard, the importance of identifying the different risk factors and mechanisms associated with injury was proposed as an effective prevention measure [14]. In football players, 60% of hamstring injuries reported were preceded by a high-speed running action [15], likely due to the rapid eccentric hamstring muscle contraction needed to perform this type of movement [16]. The failure of the tissues to tolerate the forces applied during running has also been proposed as another mechanism of hamstring strain injury [17]. Although there are no data for professional football referees, current information suggests that hamstring muscle weakness may be a main factor for the development of hamstring injury in professional football refereeing. A high physical fitness level, specifically by maintaining appropriate muscle strength in the lower limbs, may be a key protective factor against injury [18].

The Coronavirus Disease 2019 (COVID-19) pandemic has been a main disruption for professional football leagues [19], although the impact in football is minor when compared to the toll paid in human lives [20]. During the first wave of the pandemic, the health authorities and governments of most European countries imposed severe confinement measures to diminish the spread of the SARS-CoV-2 virus, which resulted in sports training and competition being suspended [21]. In Spain, home confinement entailed a strict quarantine that prohibited all individuals from practicing any form of exercise outside of their own homes to be effective from 15 March 2020, and for a total period of 8 weeks [22]. Home isolation had a severe impact on athletes because training and competition in indoor and outdoor sports facilities were suspended. During confinement, fitness trainers from the Royal Spanish Football Federation (RSFF) provided training programmes for professional football referees to be performed at home, and organised video conferences to recommend strategies to maintain referees’ physicality during this period. Due to the positive evolution of the pandemic in Spain, the government decided to alleviate confinement conditions on 11 May 2020. After this date, the referees were able to visit sports facilities and perform exercise outdoors and they returned to their habitual specific and functional training routines. Similar to professional football players, Spanish football referees had to retrain after the end of home confinement to recover their steady-state physical performance, as the national football league was set to resume by June 8 to complete the 11 fixtures remaining to finish the season [22].

Hence, professional Spanish football referees were confined at home for 8 complete weeks, and then had 4 weeks of retraining before the resumption of the Spanish football league. Similar to professional football players, professional football referees underwent a congested competitive scenario after the resumption of the competition; it entailed the completion of 11 fixtures in less than six weeks. To the authors’ knowledge, no previous investigation has offered information about the way home confinement produced detraining effects in professional football referees and how physical condition was regained during the retraining and competitive periods. To date, only one investigation has shown that hamstring muscle strength in football players was progressively reduced as the home confinement progressed in duration [23]. That investigation showed that players suffered muscle weakness during home confinement despite training at home with specific exercises to maintain muscle strength in all the structures of the lower limbs. However, it is unknown if the retraining period before the resumption of the football league was effective to diminish the detraining effect of home confinement. For this reason, the aim of the present investigation was to assess changes in eccentric hamstring muscle strength during football competition suspension/resumption due to the COVID-19 pandemic in professional football referees. This information would be key for understanding the magnitude of muscle weakness that professional referees underwent during home confinement due to COVID-19, but also the capacity of muscle strength retraining once the confinement was alleviated. We hypothesised that home confinement due to COVID-19 pandemic would induce lower eccentric hamstring muscle strength in professional referees.

## 2. Materials and Methods

### 2.1. Participants

A total of 23 male professional football referees volunteered to participate in this prospective observational study. Participants were recruited by phone calls and emails and all of them pertain to the refereeing section of the RSFF. Among these, only a group of 21 referees (mean ± standard deviation (SD), age: 33.4 ± 5.1 years; height: 182.4 ± 5.0 cm; body mass: 75.1 ± 4.4 kg; weekly training time: 7.0 ± 0.5 h) completed all testing, consisting of measurements of eccentric hamstring muscle strength at four time-points. As inclusion criteria, participants had to be free of musculoskeletal injuries with no presence of delayed onset muscle soreness in the day of the testing and they had to be active referees during the 2019–2020 season. Two referees were excluded from the study because they did not complete the four measurements. All participants were fully informed of any risks and discomforts associated with the experiments before giving their informed written consent to participate. The study protocol was reviewed and approved by the Institutional Review Board of the European University of Madrid (code: 16/2019/CEICEGC), in accordance with the latest version of the Declaration of Helsinki.

### 2.2. Experimental Protocol

In this study, all referees were exposed to the same experimental protocol. This investigation aims to provide data about changes in eccentric hamstring muscle strength during football competition suspension/resumption due to the COVID-19 pandemic in professional football referees. The first measurement of eccentric hamstring muscle strength was performed on 28 March 2020, when referees had undergone home confinement for 13 days (established as the “confinement” measurement). During confinement, the staff of the RSFF provided personalized training programmes and organized individual and group video-based activities to reduce the potential detraining effects. The training program included six training sessions per week and included strength-based activities with body loads, proprioception activities, and endurance-based exercise (e.g., treadmill running and indoor cycling). Home training protocols were adapted to the conditions of each referee’s residence. Training sessions were intended to produce exercise duration and rating of perceived exertion aligned with referees’ conventional in-season training. The second measurement was performed on 18 May 2020, one week after the measures of home confinement were alleviated (“post-confinement”). The third measurement was performed on 7 June 2020, one-to-four days before the resumption of the national football league (“pre-competition”). This entailed a retraining period of 4 weeks. The fourth measurement occurred on 22 July 2020, three days after the last fixture of the professional football competition was completed (“post-competition”).

### 2.3. Measurement of Eccentric Hamstring Muscle Strength

At all of these four time-points, eccentric hamstring muscle strength was measured using video-recordings of the Nordic hamstring test which were analysed afterwards using a video-based method [24]. For this measurement, referees performed a standardised 15-min warm-up consisting of 10 min of pedalling on a stationary bike and 5 min of dynamic stretching of the lower limb muscles. Afterwards, each referee adopted a kneeling position on a yoga mat with hands crossed over the chest and from this position started leaning forward with full extension of the back and hips to perform the Nordic hamstring exercise [25]. Referees had been previously familiarised with this test and were told to perform the Nordic hamstring exercise in a slow and controlled manner. The break angle in this test was defined as the angle between the starting position and the line joining the right femoral condyle and the right greater trochanter position when the referee could no longer withstand the force of the fall (i.e., the break-point;) [26]. Once the break point was obtained, the referees used their arms to land on the yoga mat and repeated the test after 2 min of passive recovery. This exercise was performed twice bilaterally and twice with each limb and an average value was obtained for analysis. All the trials were videotaped with a smartphone placed 3 m away on the right side of the player, 0.5 m above the floor and with the high-speed camera set at 240 frames per second. Later, the break angle was measured with a smartphone application (Nordics, My Jump Lab, Madrid, Spain) and the hamstring muscle strength was calculated by using the break angle and participant’s body mass and body height. Nordic hamstring measurements were obtained at the same time of the day and after a rest day at all four-time points of measurement and participants were encouraged to withstand the fall as much as possible during the Nordic exercise test. In addition, to reduce the interference of uncontrolled variables, all the referees were instructed to maintain their habitual lifestyle and normal dietary intake before and during the study.

### 2.4. Statistical Analysis

Means ± standard deviations were calculated from the raw database to compare eccentric hamstring muscle strength at four time-points. There were no missing data during the collection of data. A one-way analysis of variance (ANOVA) for repeated measures was used to determine the main effect time and Bonferroni post-hoc tests were applied to detect differences in pairwise comparisons. Cohen’s effect sizes (ES) were also calculated in all pairwise comparisons with the measurement obtained during home confinement to estimate the magnitude of change for strength increase in the remaining measurement time points.

## 3. Results

**Figure 1:**
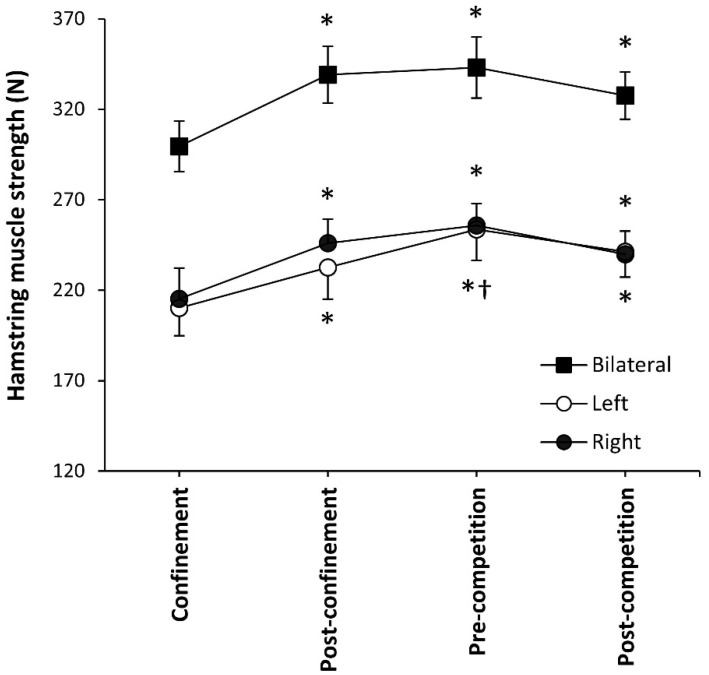
Eccentric hamstring muscle strength in professional football referees during football competition suspension/resumption due to the COVID-19 pandemic. (*) Different from measurement during confinement at *p* < 0.05. (†) Different from measurement post-confinement at *p* < 0.05.

Figure 1 depicts the changes in eccentric hamstring muscle strength during the confinement, after the confinement, and before and after the resumption of football competition for all 21 referees included in the study. The fluctuations in this variable presented statistical significance when analysed for the bilateral measurement (F = 5.818; *p* = 0.004) and for the unilateral measurements in the left (F = 10.080; *p* < 0.001) and right limb (F = 5.881; *p* = 0.002). During confinement, referees presented the lowest value of eccentric muscle strength for the bilateral and unilateral measurements, which were lower than in the remaining measurements (*p* < 0.050). In comparison to the measurement during confinement, bilateral eccentric muscle strength increased by 13.2 ± 3.7% (*p* = 0.001, ES = 2.8) in the post-confinement trial and remained stable in the pre-competition (*p* = 0.001, ES = 3.1) and post-competition measurements (*p* = 0.014, ES = 2.0). The unilateral measurements followed a similar pattern; in the post-confinement measurement muscle strength increased by 10.6 ± 1.9% in the left limb (*p* < 0.001, ES = 1.6) and by 14.3 ± 4.9% in the right limb (*p* = 0.002, ES = 2.3) with respect to the confinement. Hamstring muscle strength was further increased before the onset of the competition in the left limb (+9.1 ± 3.5%; *p* = 0.013, ES = 3.0) although the change did not reach statistical significance in the right limb (+4.0 ± 4.2%, *p* = 0.328). After the competition, there was a slight reduction in hamstring muscle strength although without statistical significance with respect to the pre-competition measurement. Still, eccentric muscle strength was higher in the left and right limbs at any time point with respect to the measurement performed during home confinement (ES from 1.6 to 3.2).

## 4. Discussion

The aim of the present investigation was to assess changes in eccentric hamstring muscle strength during football competition suspension/resumption due to the COVID-19 pandemic in professional football referees. Although there are no data on eccentric muscle strength prior to the onset of home confinement due to the COVID-19 pandemic, the fluctuation in this variable after home confinement suggests that the quarantine conditions produced detraining effects associated with hamstring muscle weakness. As happened in football players [23], the exercise routines performed at home were insufficient to prevent a potential decrease in eccentric muscle strength. A recent investigation indicates that referees’ physical performance varies across time, mostly due to the effect of training and match refereeing [27]. Hence, the likely detraining effect during home confinement shown in the current investigation was associated with the lack of competition and with the fact that the conditions of home training during confinement for most referees impeded performing high-intensity accelerative and running actions.

This investigation is innovative because it presents, for the first time, that the detraining effects of home confinement were resolved once the referees were allowed to train without restrictions. In this regard, bilateral and unilateral eccentric hamstring muscle strength increased in the post-confinement measurement which occurred only 1 week after home confinement was suspended. In addition, eccentric hamstring muscle strength slightly increased by the pre-competition measurement, suggesting that the retraining period allowed from the end of home confinement to the onset of the football national league was sufficient to resolve hamstring muscle weakness. Perhaps, the high adaptability of the hamstring muscle to different training interventions allowed regaining hamstring muscle strength in a period of 4 weeks [28,29,30]. For example, Mendiguchia and co-workers found improvements of 13% in eccentric hamstring strength after 7 weeks of ‘neuromuscular training’ emphasising eccentric hamstring exercises. Moreover, there was a non-significant decrease in hamstring muscle strength after the end of the competition. As has been found in elite young football players [31]. the accumulation of matches together with insufficient recovery time between games produce muscle fatigue that affects hamstring muscle strength. In addition, the need for travelling for match refereeing and the preparation/recovery times before/after each match reduced the time devoted to training in this sample of professional football referees.

### 4.1. Limitations

The current investigation has several limitations. First, football referees were confined at home for eight weeks and they had four weeks to retrain before the resumption of their refereeing activities. The changes in eccentric hamstring muscle strength may be different in situations that involve home confinement or inactivity with longer/shorter durations. Second, although all the referees were confined at home for the same duration, the type of exercise activities that they were able to perform during home confinement was slightly different depending on the equipment referees had available (e.g., aerobic exercise was performed with indoor cycling or running on a treadmill). Last, the sample included only male professional referees and the results should not be translated to other groups of referees.

### 4.2. Practical Applications

Today, COVID-19 is still a serious respiratory illness with high likelihood of contagion even with the presence of several vaccines and a high proportion of the population vaccinated in most developed countries. Hence, it is likely that home quarantine is needed for some athletes that have been infected with the SARS-CoV-2 or for those who have been in contact with an infected individual. Although the first objective during home confinement is to overcome the infection and the period of transmission, home training should be informed for those with slight symptoms to avoid detraining, particularly for muscle strength. To this regard, strength-based activities with body loads may be insufficient to avoid muscle weakness and the use of weights may be necessary to maintain muscle strength.

## 5. Conclusions

In summary, the results of this investigation suggest that 8 weeks of home quarantine due to COVID-19 produced hamstring muscle weakness in Spanish professional football referees. The exercise routines performed at home were not effective to prevent a detraining effect in hamstring strength, but football referees rapidly regained hamstring muscle strength once they were allowed to train in sports facilities. A period of ~4 weeks was sufficient to overcome the detraining effects of home confinement on bilateral and unilateral eccentric hamstring muscle strength as determined by the Nordic exercise test.

## Data Availability

The data presented in this study are available on request from the corresponding author.
